# Lower Limb Biomechanics during the Golf Downswing in Individuals with and without a History of Knee Joint Injury

**DOI:** 10.3390/bioengineering10050626

**Published:** 2023-05-22

**Authors:** Zi-Jun Lin, Yi-Chien Peng, Chun-Ju Yang, Chung-Yuan Hsu, Joseph Hamill, Wen-Tzu Tang

**Affiliations:** 1 Graduate Institute of Athletics and Coaching Science, National Taiwan Sport University, Taoyuan 33301, Taiwan; 2 Physical Education Office, National Cheng Kung University, Tainan 70101, Taiwan; 3 Center of Traditional Chinese Medicine, Division of Chinese Acupuncture and Traumatology, Taoyuan Chang Gung Memorial Hospital, Taoyuan 33378, Taiwan; 4 Biomechanics Laboratory, University of Massachusetts, Amherst, MA 01003, USA

**Keywords:** 3D motion, driver, kinematics, kinetics

## Abstract

Although prevention is better than treatment, after a knee injury occurs, the adjustment of the movement technique back to the posture before the injury and the restoration of accuracy is very important for professional and amateur players. This study aimed to compare the differences in lower limb mechanics during the golf downswing between those with and without a history of knee joint injury. A total of 20 professional golfers with single-digit handicaps were recruited for this study, 10 of whom had a knee injury history (KIH+), while another 10 players were without a knee injury history (KIH−). From the 3D analysis, selected kinematic and kinetic parameters during the downswing were analyzed using an independent samples *t*-test with a significance level of α = 0.05. During the downswing, individuals with KIH+ exhibited a smaller hip flexion angle, smaller ankle abduction angle, and larger ankle adduction/abduction range of motion (ROM). Moreover, there was no significant difference found in the knee joint moment. Athletes with a history of knee injury can adjust the motion angles of their hip and ankle joints (e.g., by avoiding excessive forward leaning of the trunk and maintaining stable foot posture without inward or outward rotation) to minimize the impact of changes in their movement patterns resulting from the injury.

## 1. Introduction

Inappropriate swing movements will not only affect hitting quality, but also increase the incidence of sports injuries. The golf swing is a complex movement, requiring the coordination of all of the parts of the body in order for it to be executed skillfully [1]. Although people believe that golf is a non-strenuous sport, the skeletal muscles exert a great force during the swing possibly leading to the occurrence of chronic injuries [2,3,4]. Although injuries of the lower limbs are not as common as injuries of the upper limbs and back, some studies found that the former accounts for 18% of the injuries from playing golf [5]. For professional golfers, the injury rate of the lead leg is as high as 7% [6].

Due to the specificity of this sport, players are required to strike the ball precisely on the target, which can be physically demanding [7]. It was observed that prolonged fatigue will cause unstable movements and mistakes, which may lead to an increased risk of injury resulting in a greater burden on the knee joints during the swing [8,9,10]. Previous research found that professional golfers were more vulnerable than amateurs to knee joint injury of the lead or left leg [6]. For highly trained professional and amateur golfers, compensatory movements resulting from injury may alter their original swing posture and, consequently, affect the quality of their swing. Therefore, it is crucial to identify which movements have changed for the athlete after injury and adjust their posture accordingly to restore the accuracy of their swing [11]. The knee joint plays an important role in golf [12,13]. Because of the structure of the knee joint, most movements are limited to the sagittal plane. However, in the golf swing, it is necessary to rotate through knee joint support for lower limb stability; therefore, the incidence of injuries is very high [6,14].

Exploring knee joint changes as an index during the golf swing through sports biomechanics allows for the accurate quantification and judgement of swing characteristics [15,16]. The kinematic and kinetic parameters of the knee joint measured using a motion analysis system are often utilized to compare swing skills between professional and amateur players [17] and to evaluate the prevention of sports injuries [18,19]. The movement stages of the golf swing can be divided into three parts: the backswing, downswing, and follow-through stages [20]. During the downswing stage, the center of pressure shifts to the lead foot and is completed when the club is aligned vertically to the ground in the downswing. The maximum GRF (which is caused by the slowing down of the downward motion of the body) also occurs slightly after this. The peak joint moments generally occur before this instant as well [21,22]. The factors causing knee joint injury involve the knee joint flexion and internal rotation angles [23]. However, prior studies were focused on the related characteristics of the knee joints of non-golfers to infer the risk of injury to the knee joints.

Previous research has reported that knee joint injuries from playing golf are mainly caused by the tibial rotation of the lead leg [17,24]. Research on the effects of the opening and closing of the left leg stance on the moment of the frontal plane of the knee joint in the swing process of healthy players [8] has shown that the posture of the lead leg is related to the adduction load of the knee joint. In the past, it was found that the activation state of the erector spinae was the greatest when using the driver for the swing and that the tee-off strategy might also cause a risk of injury. Previous research has also found that the ground reaction force when using a driver is higher than when using an iron club, as a wood club has a higher moment of inertia (MOI), which requires more force to swing [25]. This may also increase the load on the body and lead to an increased risk of injury [26]. In addition, compensatory movements may occur due to fatigue in golfers, resulting in subtle changes in the ankle joint angle and leaving the power chain in the knee joint [27]. This may lead to an increased load on the knee joint and an increased incidence of knee injuries. Therefore, there may be compensatory movements in the movement techniques of lower limb joints (knees and ankles) in the population with a history of knee joint injury, though there is no relevant research to date for this population. Improving the athletic performance of golfers should be considered an important aspect. At present, there is no study comparing the swing movement of players who have a history of knee joint injury with that of players’ said history in three-dimensional kinematics. Such a study could provide the characteristics of the lower limb movement technique and load after a history of knee joint injury and determine the essential key points and suggestions for golfers who recover from injury to improve their swing technique.

Therefore, the purpose of this study was to compare the axial angular displacement and moment of each lower limb joint of the lead or left leg for a right-handed golfer during the swing between two groups of highly skilled golfers with and without a history of knee joint injury. We sought to understand the posture differences in peak angle and peak moment. It is our hope that the results will serve as a reference for those with a history of knee joint injury to make corrections and improvements in their movement techniques.

We hypothesized that the group without a history of knee injury would exhibit smaller joint angles and greater knee joint moments during the golf swing. When injuries cause issues, it is common for the golfer to introduce compensation motions. If the knee is where the injury occurred, this compensation could actually change the way the golfer moved their ankle joint.

## 2. Materials and Methods

### 2.1. Participants

A total of 20 professional golfers with single-digit handicaps were recruited in this study, 10 of whom exhibited a chronic knee injury history resulting from playing golf (KIH+) (average age: 21 ± 3.2 years, average weight: 66.8 ± 11.2 kg, and average height: 166.3 ± 4 cm) while another 10 players were injury-free (KIH−) (average age: 22.8 ± 2.5 years, average weight: 72.4 ± 15.3 kg, and average height: 165.2 ± 9 cm). Sample size calculations (G*Power, version 3.1.9.7) revealed that a sample of twenty participants would be sufficient to detect a difference between groups, with a statistical power of 1 and Type I error probability, associated with the test of the null hypothesis, 0.05. All of the participants were right-hand dominant, that is, their lead leg was the left leg. They had not experienced lower limb musculoskeletal injury or nervous system injury six months prior to the study implementation. The participants signed an informed consent form before the start of the experiment. The study was approved by the relevant institutional review board, in line with the principles of the World Medical Association Declaration of Helsinki, and informed consent from all of the participants was obtained.

### 2.2. Equipment

A motion capture system (Motion Analysis, Santa Rosa, CA, USA) with 11 Eagle digital cameras was used to capture reflective marker balls for three-dimensional marker tracking [28]. The sampling frequency was set at 200 Hz. A Helen Hayes marker set, a built-in module in the motion analysis system, was used to attach 32 markers [29,30] with an additional 8 markers attached to the grip, club, and club head and 1 marker on the golf ball, for a total of 41 markers, to capture the participants’ movement patterns during the swing. The participants stood with their lead leg on the force plate (BP400600; AMTI, Newton, MA, USA) to hit the ball. The force plate can measure forces in three axes (Fx, Fy, Fz). The sampling frequency of the force platform was set at 1000 Hz. In this experiment, the participants used golf shoes for the experiment and a driver for the swing test.

### 2.3. Protocols

**Figure 1:**
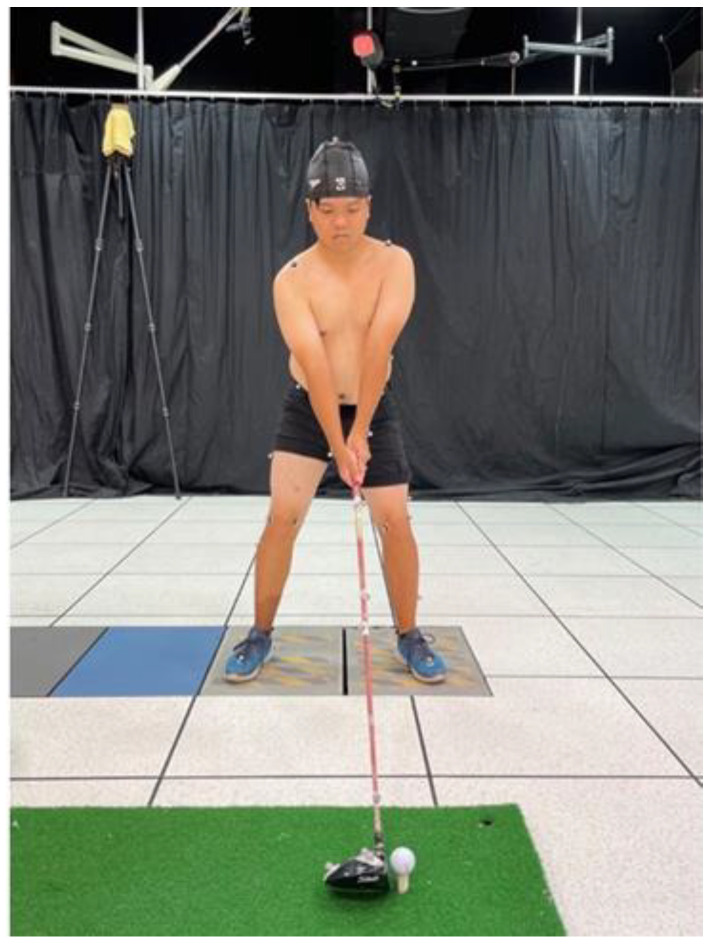
Experimental set-up showing one subject standing with his left foot on a force plate while adhering using Helen Hayes reflective marker dots.

The participants first filled out an injury survey form. After confirmation, they carried out dynamic warm-up static stretching [31] for 10 min. Markers were placed on various body parts including the head, shoulders, elbows, wrists, anterior and posterior superior iliac spine, lateral thigh and shank, lateral femoral epicondyle, lateral malleoli, the dorsum of the foot at the metatarsophalangeal joint, and the heel of the foot the swing was then completed five times. They were instructed to swing and hit the ball with the same effort as in a real game scenario during the test. The participants were required to inform the researchers of any reason (e.g., leg slip, out of balance) of a swing that did not represent a complete golf swing in which case the trial was redone. Each participant completed five trials, and the shot with the best data quality and participant response was chosen for analysis. The quality of the reconstructed 3D data and participants’ feedback was used to determine the best shot [18]. Figure 1 shows an example of data collection in which the participants were equipped with all of the markers and golf-specific training aids as shown.

### 2.4. Data Analysis 

Cortex motion analysis software (Cortex 7.1; Motion Analysis Corp., Santa Rosa, CA, USA) was used to mark the trajectory of the three-dimensional lower limb kinematics and kinetics during the downswing stage. The selected data were filtered using a Butterworth fourth-order low-pass filter to remove high-frequency noise, and the cut-off frequency was set at 6 Hz [32]. 

The participants’ general information (height, weight, and limb segment parameters) was inputted to construct a human body calculation model with body segments as rigid bodies, and relative angles were obtained using the center of the joint as a fixed point. These models were created using information obtained from the attached markers, ground reaction forces, and anthropometric data. The kinematic model involved identifying the rotation center of each lower extremity joint (hip, knee, and ankle), establishing an anatomical reference system for the thigh and shank segments, and calculating the angles of the hip, knee, and ankle joints using the Euler angle method [33,34]. The kinetic model of the knee joint was based on the inverse dynamics technique, which took into account the mass, center of mass, and moment of inertia of the foot and shank from previous anthropometric data [35]. The joint net moments were then calculated using the Newton–Euler equation [36].

The kinematic changes in the knees and ankles at the downswing stage (from the peak of the backswing to the instant of ball contact) were calculated. The joint displacement angles, range of motion (ROM), and joint moments of the lower limbs in three axial directions in the swing process were also calculated [21] and the average value of each participant’s five complete swing movements was determined. The collected data were processed by a program written in MATLAB (Version R2015b; The MathWorks Inc., Portola Valley, CA, USA).

### 2.5. Statistics

The statistical analysis and calculations were performed using SPSS statistical software (Version 22.0; IBM Corp., Armonk, NY, USA). The results were expressed as the mean ± standard deviation (SD). Normality was evaluated for each of the dependent parameters utilizing the Shapiro–Wilk test. The peak values and ROM of the kinematics and kinetics of the hip, knee, and ankle joints of the lower limbs in different anatomical directions (sagittal, frontal, and transverse planes) in the swing process were examined and compared between the KIH+ and KIH− groups using an independent samples *t*-test. The statistical significance level of this study was set at 5%. Cohen’s value was calculated to assess the effect size [37].

## 3. Results

Table 1 shows the peak angle and maximum ROM or displacement angles of the hip, knee, and ankle joints of all the participants in each anatomical direction (sagittal, frontal, and transverse planes), with the lead leg at the downswing stage. Significant differences in hip flexion, ankle abduction, and ankle adduction/abduction ROM were found between the KIH+ and KIH− groups through the independent samples *t*-test. 

The kinetics results are shown in Table 2. Through the independent samples *t*-test, no significant differences were found in the knee joint moment between the KIH group and the KIH− group.

Through the independent samples *t*-test, it was however found that the KIH+ group had a smaller hip flexion angle and ankle abduction angle, but a larger ankle adduction and abduction ROM than the KIH− group. The statistical results indicated that there was no significant difference in the knee joint moment between the two groups.

## 4. Discussion

The purpose of this study was to explore the influences of a lower limb injury history on the lead foot during the golf downswing. The results indicate that compared with the KIH− group, the KIH+ group had a larger hip flexion angle, smaller ankle abduction angle, and larger ankle adduction and abduction ROM. The results partially conform to the research hypothesis.

In the biomechanics literature, there has been little relevant sports science research on the effects of injury history on movement strategies. The results of this study can effectively provide information on relevant sports biomechanical parameters and explore the trends of swing pattern changes and differences in those with and without a history of knee joint injury. Previous studies reported the golf knee load ranged from being similar to a stair descent, to tennis serving or jogging, and concluded that the peak load generated in the golf swing was not enough to cause immediate injury and that the risk of knee joint injury was caused by repeated swings over a prolonged period [38]. On the other hand, our studies showed the KIH− group had smaller values for the axial rotational moment than other research with PGA participants during the downswing (knee external rotation moment: −0.24 ± 0.26 vs. −0.32 ± 0.11 Nm/kg, respectively) [2]. It is suggested that the KIH− group should avoid prolonged swing training that can lead to overuse injuries. Adequate rest is crucial. Moreover, golfers should engage in lower limb strength training to enable their bodies to adapt to the loads generated during the swing and thus reduce the incidence of sports injuries [39]. More interestingly, our study showed the KIH+ group values to be even smaller (−0.09 ± 0.06 Nm/kg) for the axial rotational moment than the KIH− group and other research during the downswing. This may possibly be due to a compensation pattern being adopted after injury for the KIH+ group. Previous research has found that hitting the ball closer to the body reduces the peak internal rotation torque of the knee joint, while hitting the ball farther away from the body reduces the peak adduction torque of the knee joint. For patients with knee arthritis, they may prefer hitting the ball farther away from the body to reduce the knee joint load [14].

However, through the descriptive statistics used in this study, it was found that the deviations in the ankle adduction/abduction angle ROM at the downswing stage in the KIH− group were lower than those in the KIH+ group. Therefore, when there was no knee joint injury, the player’s hitting strategy in the transverse plane was identified as being more stable with less ROM. Previous studies have suggested that individuals with knee osteoarthritis may prefer hitting the ball from a farther distance [14]. Therefore, in this study, it was hypothesized that the KIH+ group would exhibit a forward shift in the center of mass, leading to a greater hip flexion angle and an increased ankle adduction/abduction ROM to maintain knee stability. On the other hand, the KIH+ group would exhibit greater maximum hip flexion and ankle adduction/abduction ROM during the downswing, which could result in unstable ankle movements (adduction/abduction ROM).

This study demonstrates that the KIH+ players used more hip flexion and ankle adduction/abduction angle ROM than the KIH− group. A previous study showed that the range of knee axial rotation could result in poor contact locations at the edges of the polyethylene surface of a total knee arthroplasty (TKA) in golfers, potentially leading to chronic pain and prosthesis damage [40]. Past studies have reported that the magnitudes of knee axial rotation at each phase of the swing varied between participants. However, a clear pattern of tibial external rotation during phases of the backswing followed. A large amount of internal tibial rotation during the downswing was observed, which is stressful for the knee joint’s internal rotation [17]. Moreover, the KIH− group had similar hitting strategies. Thus, from the information above, we can effectively understand the “risky” movement pattern for a knee injury, correct the hitting strategy, and avoid the risk of injury. Moreover, previous studies have utilized varying ankle angles, including self-selected, 0° with the toes pointing forward, and 30° of ankle external rotation, and stance widths (self-selected, narrow, and wide) to carry out the swing. It was found that a relatively wide stance at 30° could reduce the peak moment of knee adduction of the lead leg without affecting the swing speed [41]. In previous studies, the effects of the lead leg position at 0° (perpendicular to the target line) and external rotation at 30° (rotated from 0°) on the knee joint moment during the swing have been compared. These studies have shown that the stance of rotating the lead foot externally by 30° during the swing can reduce the knee joint adduction moment [8]. Moreover, different abduction angles of the ankle joint can also affect the load on the knee joint [8]. In this experimental design, the golfer’s stance was not altered to avoid affecting their swing technique. This study suggested that the leg’s initial position could be considered to move external rotation to avoid excess leg abduction and knee moment axial load. It also implied that changes in the hip and ankle angles may affect the knee joint moment and pose a high potential risk, highlighting the need for preventive training.

Sports injuries often affect sports performance [42], and many excellent athletes’ sports performance is not as good as before their injuries occurred; thus, they require physical therapy [43] and a movement technique diagnosis through sports science [15,18,19] to alleviate sports injury and restore their movement techniques to the level before injury. In this study, we found that people with a KIH+ may exhibit compensatory postures, such as a smaller ankle abduction angle, larger hip flexion, and greater ranges of motion for ankle adduction and abduction in the distal segments of the foot on the ground. It is recommended to pay closer attention to the motion angles of the hip flexion and ankle adduction/abduction joints. This includes avoiding excessive forward leaning of the trunk and maintaining a stable foot posture, without ankle adduction or abduction. during recovery exercise after injury. Based on our findings, we conclude that it is necessary for golf players to undergo long-term biomechanical testing, which can serve as a reference for their sports technical characteristics and improve their performance after injury. By focusing on the changes in hip and ankle angles, as revealed by our study, preventive measures can be taken to avoid sports injuries.

## 5. Conclusions

This study suggests that athletes who have recovered from knee injuries should be mindful of their hip flexion angle, ankle abduction angle, and their ROM for ankle adduction and abduction in order to minimize the impact of any posture changes resulting from the injury. In addition, it is also suggested that it is necessary for golfers to undergo long-term sports biomechanical testing, which can be used not only to prevent sports injuries, but also to recover from injuries through these data and to identify problematic feature points to improve their sports performance.

## Figures and Tables

**Table 1:** Differences in lower limb kinematics between the KIH+ and KIH− groups.

Degrees (°)	KIH+	KIH−	t-Value	*p*	Effect Size
Hip flexion	31.15 ± 15.29	17.44 ± 8.52	2.47	0.023 *	1.1
Hip extension	1.84 ± 10.79	−5.78 ± 7.54	1.83	0.083	0.8
Hip flex/ext ROM	29.3 ± 7.17	23.22 ± 7.38	1.86	0.078	0.8
Hip adduction	8.29 ± 5.88	7.40 ± 3.65	0.40	0.691	0.1
Hip abduction	−31.94 ± 10.58	−29.54 ± 6.16	−0.62	0.543	0.2
Hip add/abd ROM	40.23 ± 9.29	36.95 ± 8.24	0.83	0.414	0.3
Hip internal rotation	43.98 ± 17.31	42.11 ± 10.68	0.29	0.774	0.1
Hip external rotation	−27.07 ± 11.92	−29.99 ± 10.10	0.58	0.563	0.2
Hip ir/er ROM	71.06 ± 18.48	72.10 ± 11.66	−0.15	0.882	0.0
Knee flexion	42.27 ± 11.12	37.06 ± 7.44	1.23	0.234	0.5
Knee extension	10.29 ± 11.47	5.61 ± 5.50	1.16	0.26	0.5
Knee flex/ext ROM	31.97 ± 9.19	31.44 ± 8.03	0.13	0.891	0.0
Knee adduction	−5.73 ± 3.43	−4.77 ± 4.92	−0.50	0.617	0.2
Knee abduction	−10.27 ± 7.09	−5.00 ± 13.26	−1.11	0.282	0.4
Knee add/abd ROM	4.84 ± 7.61	0.23 ± 12.00	0.95	0.35	0.4
Knee internal rotation	−20.26 ± 9.56	−22.32 ± 8.35	0.51	0.615	0.2
Knee external rotation	−37.92 ± 8.39	−41.27 ± 6.70	0.98	0.337	0.4
Knee ir/er ROM	17.65 ± 8.73	18.95 ± 7.21	−0.36	0.722	0.1
Ankle plantarflexion	20.59 ± 5.00	17.96 ± 2.96	1.42	0.171	0.6
Ankle dorsiflexion	−3.28 ± 9.54	−4.52 ± 6.26	0.34	0.733	0.1
Ankle pla/dor ROM	23.87 ± 6.44	22.48 ± 5.38	0.52	0.609	0.2
Ankle adduction	19.50 ± 4.72	20.82 ± 3.16	−0.73	0.471	0.3
Ankle abduction	10.48 ± 3.64	15.21 ± 3.12	−3.11	0.006 *	1.3
Ankle add/abd ROM	9.01 ± 3.16	5.61 ± 2.07	2.84	0.011 *	1.2
Ankle internal rotation	−1.66 ± 6.28	0.64 ± 4.75	−0.92	0.366	0.4
Ankle external rotation	−8.01 ± 6.65	−5.43 ± 4.45	−1.02	0.321	0.4
Ankle ir/er ROM	6.35 ± 3.56	6.08 ± 2.58	0.19	0.846	0.0

* Indicates significant differences.

**Table 2:** Differences in lower limb kinetics between the KIH+ and KIH− groups.

Moment (Nm/kg)	KIH+	KIH−	t-Value	*p*	Effect Size
Knee flexion	1.03 ± 0.62	1.11 ± 0.47	−0.32	0.751	0.1
Knee extension	−0.17 ± 0.25	−0.23 ± 0.26	0.53	0.598	0.2
Knee adduction	0.37 ± 0.25	0.29 ± 0.22	0.72	0.480	0.3
Knee abduction	−0.60 ± 0.26	−0.47 ± 0.55	−0.67	0.508	0.2
Knee internal rotation	0.18 ± 0.06	0.22 ± 0.07	−1.25	0.223	0.5
Knee external rotation	−0.09 ± 0.06	−0.24 ± 0.26	1.75	0.096	0.7

## Data Availability

All data are contained within the manuscript.
